# Environmentally-Induced Transgenerational Epigenetic Inheritance: Implication of PIWI Interacting RNAs

**DOI:** 10.3390/cells8091108

**Published:** 2019-09-19

**Authors:** Karine Casier, Antoine Boivin, Clément Carré, Laure Teysset

**Affiliations:** Transgenerational Epigenetics & small RNA Biology, Sorbonne Université, CNRS, Laboratoire Biologie du Développement, Institut de Biologie Paris-Seine, UMR7622, 75005 Paris, France; karine.casier@upmc.fr (K.C.); antoine.boivin@sorbonne-universite.fr (A.B.); clement.carre@sorbonne-universite.fr (C.C.)

**Keywords:** piRNA, transgenerational epigenetics, environment, transposable elements

## Abstract

Environmentally-induced transgenerational epigenetic inheritance is an emerging field. The understanding of associated epigenetic mechanisms is currently in progress with open questions still remaining. In this review, we present an overview of the knowledge of environmentally-induced transgenerational inheritance and associated epigenetic mechanisms, mainly in animals. The second part focuses on the role of PIWI-interacting RNAs (piRNAs), a class of small RNAs involved in the maintenance of the germline genome, in epigenetic memory to put into perspective cases of environmentally-induced transgenerational inheritance involving piRNA production. Finally, the last part addresses how genomes are facing production of new piRNAs, and from a broader perspective, how this process might have consequences on evolution and on sporadic disease development.

## 1. Introduction

The impact of environmental stress on gene expression has been known since the 1960s. In the presence of lactose, glucose starvation in *Escherichia coli* induces derepression of the lactose operon, allowing cells to metabolize this sugar. These gene regulation changes are maintained until glucose is added to the nutritive medium without impacting subsequent generations [1].

In animals, plants, or fungi, some stress can induce epigenetic changes that can be maintained for at least more than one generation even if the stress is removed. Early studies analyzing human populations bring to light some physiological effects of parental food intake on their children’s health, for instance, in Sweden [2], during the Leningrad siege (1941–1944) [3] or the Dutch famine (1944–1945) [4,5]. A restricted number of studies identified epigenetic modifications associated with parental stress [6]. Identification of accurate molecular associated modifications remains difficult to pinpoint, because of (1) the obvious impossibility of establishing a classical design of experiment, such as reproducibility and control experiments during several human generations, (2) past cultural and ecological factor influences [7], and (3) multiple stresses (nutritional, psychological, or social) that go along with wars and famines. Therefore, to better understand the consequences of specific stress at the epigenetic level and their possible transmission, it is critical to develop animal models.

Epigenetic inheritance is characterized by a reversible phenotypic transmission to the next generation in the absence of DNA sequence modification. This definition can be applied in an extended sense, including social learning and communication or in a tight sense, like mother to daughter cell transmission, called ‘cellular epigenetic inheritance’ [8]. In the latter, it is also important to differentiate intergenerational, multigenerational, and transgenerational inheritance. If the stress condition impacts the F0 gestating female and the F1 progeny (embryos), it will be referred to as an intergenerational effect. If the F2 progeny (embryonic germ cells) are also affected, it will be referred to as a multigenerational effect. In contrast, transgenerational inheritance corresponds to an epigenetic transmission of stress passing through the germ cells, but without direct exposure. Hence, male and non-gestating female transgenerational phenotypes can be considered in F2, whereas for gestating female, only F3 has to be considered [9].

In the past twenty years, the development of next generation sequencing (NGS) approaches led to the accumulation of convincing evidence for environmentally-induced transgenerational epigenetic inheritance dependent on DNA methylation, specific histone modification, and non-coding RNA biology. However, the precise mechanisms remain unclear, leaving open questions to be tackled. PIWI-interacting RNAs (piRNAs), a class of small non-coding RNAs that tightly repress transposable element (TE) activity in animal gonads, have also been demonstrated to induce stable epigenetic memory. In this review, we present some examples of environmentally induced transgenerational epigenetic inheritance to underscore the role of piRNAs in epigenetic inheritance and more specifically, in transgenerational epigenetic inheritance in response to environmental stress. Finally, the implications of piRNAs in environmentally-induced transgenerational inheritance in evolution and potential implication in human health will be discussed.

## 2. Evidence and Mechanisms of Environmentally-Induced Transgenerational Epigenetic Inheritance

The universal aspect of transgenerational epigenetic inheritance is now well documented in several species from different taxa, including bacteria, protists, fungi, plants, and animals [8]. About one-third of these transgenerational effects are linked to environmental factors, such as nutritional stresses (starvation or supplementation), chemical stresses (arsenic), and physical stresses (temperature, pressure, light and dark exposure).

### 2.1. Multiple Molecular Supports for Environmentally-Induced Epigenetic Inheritance

Four main types of support have been identified including (1) DNA that can be methylated on CG, CH, and CHH motifs, where H corresponds to a nucleotide other than G, (2) histones that can be modified (trimethylation of histone 3 lysine 4 (H3K4me3) associated with transcriptionally active marks and di/trimethylation of histone 3 lysine 9 (H3K9me2/3) and trimethylation of histone 3 lysine 27 (H3K27me3) associated with transcriptionally repressive marks) or replaced in the late-stage spermatids of many animals by the protamines forming a highly compact genome [10]. Defect of histone replacement during gametogenesis, called ‘histone retention’, could be associated to some infertility in men [11], (3) small non-coding RNAs (microRNAs (miRNAs), short interfering RNAs (siRNAs), PIWI-interacting RNAs (piRNAs), and transfer RNA-derived small RNAs (tsRNAs) (also known as tRNAs Fragments (tRFs)), and (4) long non-coding RNAs (lncRNAs) [12,13,14].

Two additional supports for epigenetic inheritance are possible, although evidence of environmentally-induced epigenetic inheritance is still lacking. First, protein structures involving prions or the Hsp90 chaperone protein have both been linked to chromatin remodeling factors in *Drosophila* and could be good candidates for involvement in such mechanisms (for more details, see [15,16]). Second, over the years, more than 100 RNA modifications have been described, some linked to human diseases [17,18]. Among the enzymes involved in these modifications, the m^5^C Dnmt2 RNA methyltransferase has been shown to be required in mice for epigenetic transmission of Kit and Sox9 phenotypic variants [19].

Although evidence is still lacking concerning protein structure and RNA modifications, transgenerational environmentally-induced epigenetic inheritance associated with DNA methylation, histone modifications, or non-coding RNAs has been reported. Some examples are presented in Table 1, which highlights that one stress can induce multiple epigenetic modifications. For instance, in rat sperm, vinclozolin or DDT pesticide exposure modifies DNA methylation, drives histone retention, and decreases the global number of expressed small non-coding RNAs (miRNA, piRNAs, and tsRNAs) and long non-coding RNAs, affecting expression of genes implicated in metabolism, signaling, and transcription [20,21] and is correlated with the occurrence of several pathologies [22,23,24,25]. In *Drosophila*, the elevation of temperature during development causes functional piRNA emergence associated with H3K9me3 enrichment from a particular repeated locus mimicking a piRNA cluster for over 50 generations [26] (Table 1). Such cross-talk between several epigenetic modifications is not restricted to animals. In fission yeast, the RNA interference (RNAi) machinery is necessary to direct H3K9me3 mark deposition, inducing heterochromatin formation and silencing on a centromeric transgene. This new epigenetic state, sensitive to the H3K9 methyltransferase mutant, can then be maintained up to 32 generations [27]. Taken together, H3K9me3 marks and small non-coding RNA could act together to reinforce epigenetic stability through generations thanks to a feedback loop: on the one hand, H3K9me3 leads to heterochromatin formation and recruits the RNAi machinery, and on the other hand, the RNAi machinery targets H3K9 residues and induces their trimethylation [26,27].

### 2.2. How Long Does the Imprinting of Transgenerational Epigenetic Inheritance Last?

At each generation, the totipotency of primordial germ cells is ensured by the erasure of epigenetic modifications requiring numerous genetic factors, such as DNA demethylation, histone methyltransferases, and histone demethylases [55,56,57]. However, analyses of DNA methylation patterns show that some loci escape this resetting, suggesting that antagonist mechanisms provide maintenance of some epigenetic marks [58]. In fact, the DNA methyltransferases *Dnmt3a* and *Dmnt3b*, the PRC2 complex in mice and the PRC2 complex associated with H3K27me3 marks in worms have been shown to be involved in the maintenance of DNA methylation and histone modification pattern on discrete loci [59,60,61].

Some studies have addressed this question for small non-coding RNAs. In *Caenorhabditis elegans*, the RNAi machinery provides its own regulation by targeting RNAi genes and leads to a progressive decrease of small RNA at each generation, varying from one to four generations [62,63]. Antagonist molecular mechanisms govern the duration of the small RNA transgenerational response. As for other modifications, transgenerational small RNA inheritance relies on many genes coding for RNA-dependent RNA polymerase (RdRp), Argonaute proteins, or histone methyltransferases (Reviewed in [64,65,66]). The duration of the transgenerational response can be extended due to siRNA, which constitutes a second RNAi trigger that drives tertiary siRNA production [67]. Hence, the duration of epigenetic inheritance depends on the influence of these two opposite processes, namely resetting and stimulation of small RNA production [68,69]. Disruption of the endo-siRNA pathway leads to hypersensitivity to exogenous small RNAs, suggesting competition between these RNAi pathways, probably for molecular factors, such as DCR-1, which is the unique *Dicer* ortholog in *C. elegans* [70]. In contrast, piRNA producing loci transcription is actively maintained at each generation by maternal piRNA inheritance in *Drosophila* [71,72]. Disruption of this maternal deposition is followed by a complete erasure after only one generation [26,71].

The family of small non-coding RNAs is frequently considered in transgenerational epigenetic inheritance, probably because there are responsible for endogenous gene regulation and involved in important developmental programs (Reviewed in [73]). In the particular case of piRNAs, their function in epigenetic memory associated with their role in response to environmental factors has been recently demonstrated [26,46].

## 3. piRNAs in Environmentally-Induced Transgenerational Epigenetic Inheritance

### 3.1. Overview of piRNA Biogenesis and Functions

**Figure 1:**
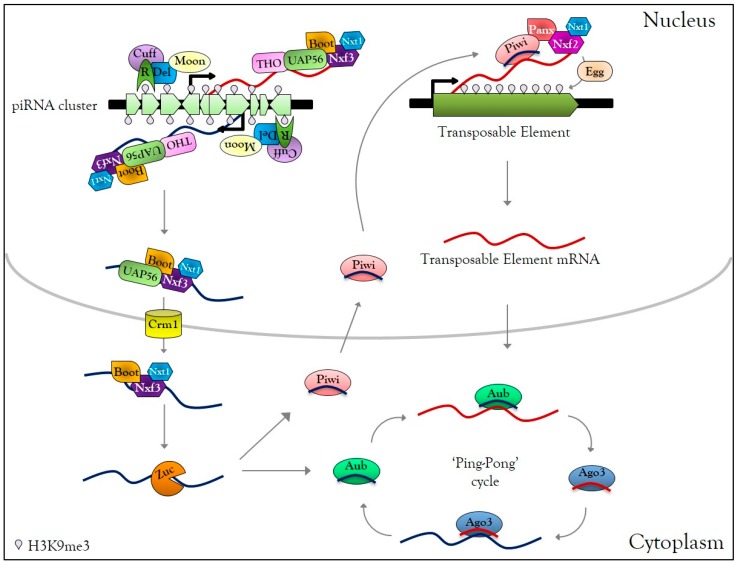
Germline PIWI-interacting RNAs (piRNA) biogenesis. In *Drosophila melanogaster* germ cells, active piRNA clusters are enriched in H3K9me3 and HP1-like protein Rhino (R), which interact with Deadlock (Del), Cutoff (Cuff). Moonshiner (Moon) interacts with Del and recruits transcription initiation factors, allowing transcription of piRNA clusters in the sense and antisense direction. Bootlegger, UAP56, the THO complex, Nxt1, Nxf3 are recruited to the piRNA cluster transcription site. The transcripts are then exported to the cytoplasmic piRNAs biogenesis site via the CRM1 exportin. In the cytoplasm, piRNA precursors are cleaved by Zucchini (Zuc) into primary piRNA and charged by Piwi or Aubergine (Aub) proteins, forming Piwi-piRISC and Aub-piRISC complexes. Piwi-piRISC is translocated into the nucleus and recognizes nascent TE transcripts due to the interaction with Panoramix, the Nxf2, and Nxt1. The association of these factors to nascent TE transcripts causes recruitment of the histone methyltransferase Eggless that transcriptionally silences TEs by local heterochromatinization. In the cytoplasm, Aub-piRISC cleaves TE transcripts into secondary piRNAs, which are loaded onto Ago3, forming Ago3-piRISC complexes. Ago3-piRISC recognizes and cleaves piRNA precursors into new secondary piRNAs, loaded onto Aub. This mechanism, known as the ‘Ping-Pong’ cycle, allows the amplification of piRISC complexes and post-transcriptional regulation of TEs.

The piRNAs correspond to a class of small non-coding RNAs interacting with the PIWI proteins (Piwi, Aub, and Ago3 in *Drosophila*), belonging to the AGO subfamily proteins, mainly active in animal gonads to protect genome integrity against TEs. In *Drosophila* germ cells, piRNAs are produced from specific heterochromatic loci, composed of numerous TE fragments, called piRNA clusters [74,75]. Those clusters are enriched in the H3K9me3 mark and Rhino HP1-like protein [76] (Figure 1). Rhino interacts first with the co-factors Deadlock and Cutoff, then Deadlock recruits the transcriptional initiation machinery via the large subunit of transcription initiation factor II A (TFIIA-L) paralog called Moonshiner, as well as Bootlegger, UAP56, the THO complex, and nuclear export factors Nxt1 and Nxf3 [76,77,78,79]. The piRNA precursor transcripts emerging from piRNA clusters are then exported by the Crm1 (chromosomal maintenance 1) exportin to the cytoplasmic piRNA biogenesis site, where they undergo a single strand hydrolysis by the Zucchini RNase to finally give rise to piRNAs [78,79,80,81,82,83] (Figure 1). In contrast to si- and miRNA, piRNAs are charged directly as single guide RNA onto PIWI proteins to form piRNA-induced silencing complexes (piRISC) [74]. After nuclear translocation, piRISC complexes recognize nascent TE transcripts and perform transcriptional gene silencing (TGS) by local heterochromatinization of TEs. This silencing requires nuclear Piwi-piRNAs that direct Panoramix, Nxf2, its co-factor, Nxt1, and the histone methyltransferase Eggless to the TE loci [84,85,86,87,88,89,90,91] (Figure 1). In the cytoplasm, mRNAs of TEs are cleaved by piRISC complexes, leading to post-transcriptional gene silencing (PTGS) of mobile elements. Cleaved-transcripts are not degraded; they are charged by PIWI protein to form new piRISC complexes, corresponding to an amplification mechanism called the ‘Ping-Pong cycle’ [74] (Figure 1). piRNA-dependent silencing occurs at each gonadal developmental stage [92], and disruption of the piRNA pathway leads to TE mobilization [93], which induces DNA damage [94,95].

PIWI protein distributions reveal that the piRNA pathway is highly conserved in animals, but they exist neither in plants nor in prokaryotes [96]. Interestingly, recent studies show that piRNAs are also found in somatic tissues of mammalian, arthropods, and mollusks [97,98,99,100]. In *Drosophila melanogaster*, however, piRNA expression is restricted to the gonads, including the germline nurse cells and the somatic follicular cells [101,102]. These findings might suggest that the ancestral capacity to synthesize piRNAs in somatic tissues (outside of gonads) has been lost in *D. melanogaster*. However, this is still subject to debate [103,104,105].

### 3.2. Role of piRNAs Outside of TE Regulation

Genomic annotations of small RNA libraries isolated from various metazoans reveal that piRNAs are not restricted to TE regulation but can also target coding genes. In *Drosophila*, piRNAs derived from the 3′UTR of *traffic-jam (tj)* matching *Fasciclin III (FasIII)* can be identified in the ovarian somatic cells (OSC) line. This finding suggests that *FasIII* is regulated by piRNAs originated from the *tj* 3′UTR [102,106]. In *Drosophila* early embryos, *nanos* (*nos*) is regulated by piRNAs. Evidence suggests that maternal *nos* mRNAs are targeted by a complex composed of piRNAs, Aub, and CCR4-NOT deadenylation factors in the soma, whereas in the germ plasm, those transcripts are stabilized by the interaction with Aub [107,108]. In Aplysia, piRNAs seem to be implicated in *CREB2* promoter methylation, a transcriptional repressor involved in long-term memory [109]. As the last example, sex determination in silkworm is dependent on the piRNA pathway that regulates *Masculinizer*, a locus required for the production of the *doublesex* female-specific isoform [110]. Taken together, these data highlight the idea that piRNAs are required in gene regulation of a broad range of cellular functions.

### 3.3. piRNAs in Epigenetic Memory

In *Drosophila*, the insertion of exogenous *lacZ* sequences into a subtelomeric piRNA cluster can functionally repress a euchromatic *lacZ* transgene in *trans* [111]. This epigenetic silencing mechanism, referred to as the *trans*-silencing effect (TSE), requires maternally but not paternally inherited piRNAs [71,112,113,114,115,116]. These results strongly suggest an active role for piRNAs in TSE and show that a piRNA cluster, without maternal piRNA inheritance, is not sufficient to promote this silencing process [112]. Involvement of piRNAs in epigenetic memory has been associated with epiallele emergence through a paramutation phenomenon in *D. melanogaster* and in *C. elegans* [71,117]. Paramutation is defined as a modification of one allele induced by another allele without DNA sequence modification and corresponds to an epigenetic conversion (for reviews see [118,119,120].

**Figure 2:**
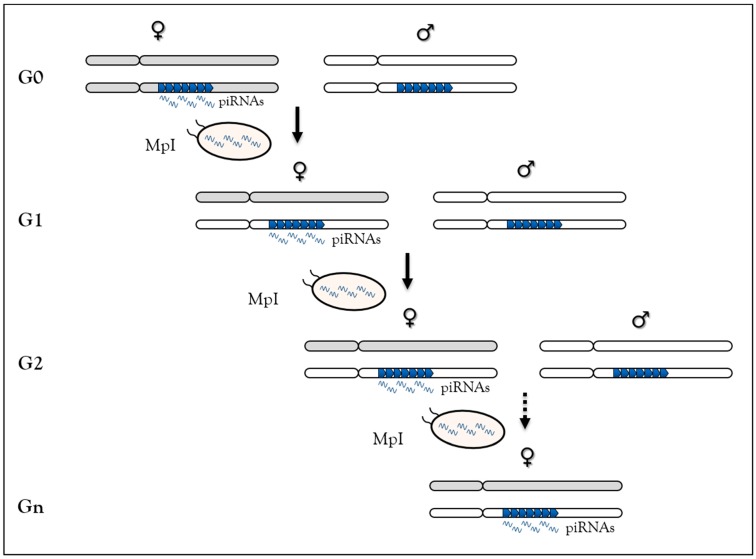
The paramutation process in *Drosophila melanogaster* relies on maternal piRNA inheritance. At G0, females (grey chromosomes) carrying the *BX2* clusters made of repeated *P-lacZ-white* transgenes (blue arrowheads) producing piRNAs are crossed to males (white chromosomes) carrying the same cluster not producing piRNA. At G1, maternal piRNA deposition in the embryo leads to activation of piRNA production from the paternal cluster allele. The newly activated cluster can activate the paternally-inherited inactive cluster in G2 due to maternal piRNA inheritance (MpI). Then, this process of activation leads to stable piRNAs production across generation.

In *Drosophila*, using a locus made of repeated *P-lacZ-white* transgenes mimicking a piRNA cluster (called *BX2* cluster), but inactive for piRNA synthesis, we have shown that homologous maternally-inherited piRNAs can stably convert this locus into an active piRNA cluster for over 200 generations [71] (Figure 2). This newly-activated cluster has the same properties as other *Drosophila* piRNA clusters. This piRNA-induced paramutation process requires molecular actors of the piRNA pathway [72] and performs TE-derived sequence control generation after generation. Finally, the reversibility of paramutation occurs when maternal piRNA inheritance is interrupted, underscoring the idea that this is an epigenetic process [26,71]. Taken together, based on sequence complementarity, these data show that maternally-inherited piRNAs are competent to initiate new piRNA production in the next generation and suggest that piRNAs are responsible for their own maintenance across generations. Two non-exclusive molecular mechanisms have been proposed to explain this case of epigenetic memory in which piRNA production requires piRNA inheritance [121]. In the first, inherited-piRNAs would induce heterochromatin formation on the piRNA cluster by leading to H3K9me3 deposition, inducing local Rhino enrichment [26]. The piRNA cluster-specific transcription machinery will then assemble at the locus to promote piRNA synthesis [77]. In the second, inherited piRNAs could trigger their production by Ping-Pong amplification from piRNA cluster precursors and TE transcripts [74,75].

In *C. elegans*, piRNAs correspond to 21 nucleotide (nt) long small RNAs with a uridine bias at the 5′-end (21U RNA) distinct from the endo-siRNAs that are 22 nt long small RNAs with a guanosine bias at the 5′-end (22G RNA) [122]. The biogenesis of nematode piRNAs depends on the Piwi protein, PRG-1 [66,123,124]. Like in *Drosophila*, transgenes are used to decipher the molecular mechanisms of germline repression. Analysis of the piRNA sensor indicates that piRNAs are required to trigger silencing through imperfect base-pairing [125,126,127]. This silencing is then transgenerationally propagated by the 22G RNA dependent on numerous factors, such as RdRp, NRDE-2, the Argonaute proteins WAGO-9/HRDE-1 and WAGO-4 and chromatin components, such as the histone methyltransferases SET-25, SET32, and HPL-2, a Heterochromatin 1 Protein (HP1) orthologue [45,128,129,130,131,132,133]. Indeed, specific H3K9 methyltransferases, SET-25 and SET-32, and endo-siRNAs have been shown to work together to promote silencing inheritance [134]. In addition, neuronal RDE-4 and germline HRDE-1 have been implicated in transgenerational regulation of endo-siRNAs and mRNAs controlling chemotaxis [135]. The authors proposed the existence of an intricate interaction between the neuronal system and the germline to control transgenerational behavior. Finally, precise dissection of the silencing mechanism determines that piRNAs lead to the production of secondary and tertiary siRNAs, necessary for full repression and leading to an epigenetic conversion form of paramutation [67].

Altogether, the piRNA silencing pathway in *Drosophila* involves piRNAs for the initiation and maintenance through generations [26,71,72], while in worms piRNAs are involved in triggering the silencing that is then maintained by siRNAs in the progeny, independent from piRNAs [124,136,137,138].

### 3.4. Heritable Stress-Induced piRNA Synthesis

In F0 mice, an early maternal separation causes downregulation of piRNA cluster 110 [139], and a Western-like diet leads to differential expression of 190 piRNAs distributed on 63 piRNA clusters [140]. In rat, a high-fat diet induces deregulation of 1092 piRNAs, and only three of them are still differentially expressed at the next generation [141]. In *C. elegans,* high temperature (25 °C) induces motif-dependent piRNA biogenesis downregulation [142]. This downregulation is associated with differential gene expression and fitness reduction, which persist at the next generation. However, in those studies, piRNA expression in subsequent generations, as well as their roles in a phenotypic response and the potential consequences on the epigenome, was not directly addressed. In the case of rat stressed with vinclozolin or DDT, differentially expressed piRNAs were identified in F3, but the mechanism and role of piRNAs were not questioned [20,21,22,23,25] (Table 1).

This year, two cases of environmental stresses inducing a genomic response dependent on piRNAs have been published in *C. elegans* and in *D. melanogaster* [26,46].

#### 3.4.1. Behavior in *C. elegans*

Wild type male or female larvae of *C. elegans* exposed to pathogenic *Pseudomonas aeruginosa* learn to avoid it and switch their feeding preference to nonpathogenic bacteria. This learning to distinguish between pathogen and non-pathogen does not correspond to a general phenomenon but rather is specific for *P. aeruginosa* [46]. Surprisingly, this behavior of avoidance can be transgenerationally inherited to the naïve F1 progeny up to the fourth generation (F4). This peculiar inheritance is completed at the fifth generation, where progeny exhibits a naïve behavior. Differential transcriptome analyses were performed on treated vs. non-treated mothers fed with pathogenic *P. aeruginosas* and their respective progeny. Moore and colleagues showed that the TGF-β ligand DAF-7, previously identified as a neuronal responder of the pathogen [143], was expressed more in specific sensory neurons of treated mothers relative to non-treated animals as well as in the F1, F2, F3, and F4 progeny, thus mirroring transgenerational avoidance [46]. The authors further show that in the *daf-7* loss of function mutant, while the mother can learn to avoid the pathogen, the F1 progeny did not receive the information of avoidance. The same pattern of inheritance was observed for mother defective for the Piwi encoding gene *prg-1*. This mutation is associated with a defect in piRNA expression in the mothers and in *daf-7* induction in the ASI neurons in F1.

Altogether, the studies indicate that transgenerational inheritance involved in the avoidance of pathogenic *P. aeruginosa* depends on the TFG-β pathway linked to the small non-coding RNAs as well as piRNA molecular partners. Therefore, piRNAs might participate in the control of specific reversible behaviors, which might have consequences on adaptive survival, providing advantages to progeny. In addition, the loss of inheritance after five generations might be considered an advantage in worms that may colonize a new environment in their natural habitat. The precise molecular basis of this resetting occurring at the fifth generation was not addressed but might be reminiscent with previous studies [63].

#### 3.4.2. Heat Response Induces de novo piRNAs

**Figure 3:**
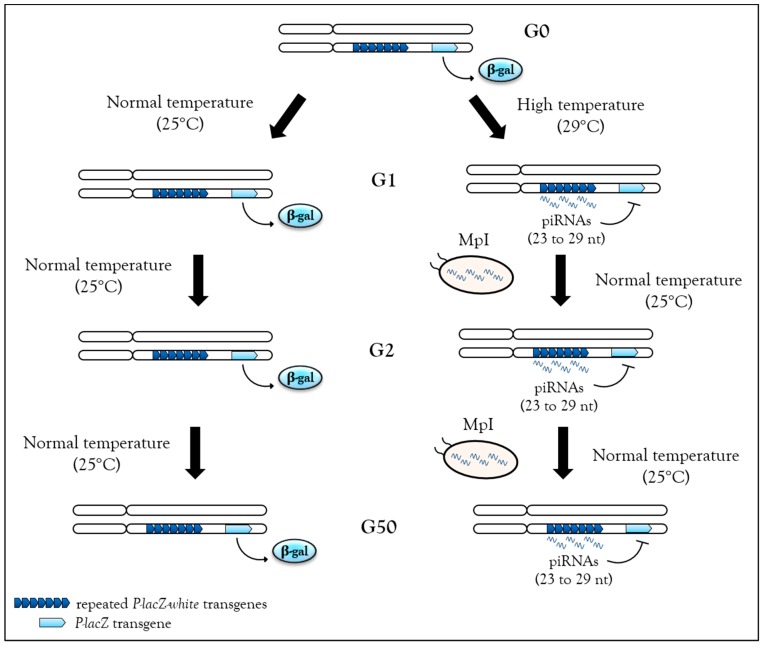
Stable, heritable stress-induced de novo piRNAs. In flies, the *BX2* cluster, a sequence made of repeated *P-lacZ-white* transgenes (dark blue arrowheads), is capable of producing piRNA after one generation at 29 °C, then these piRNAs can functionally repress a homologous *P-lacZ* sequence (light blue arrowheads) in *trans*. This silencing capacity is maintained at the second generation after return to normal temperature (25 °C) and then up to 50 generations. Hence, when stress is removed, piRNAs can be self-maintained by maternal piRNA inheritance (MpI) at each generation.

Recently, high temperature during development (29 °C instead of 25 °C) has been shown to be sufficient to induce de novo piRNA production and heterochromatin associated with H3K9me3 and Rhino enrichment from the *BX2* cluster in *Drosophila* [26]. One generation raised at 29 °C is sufficient to activate piRNA production from the *BX2* cluster, and once this production is started, it can be maintained over 50 generations even after return to normal temperature (25 °C) (Figure 3). These piRNAs can functionally silence homologous *lacZ* reporter transgenes, a phenomenon referred to previously as TSE. Hence, heat stress is able to induce piRNA production from a locus comprised of repeats. Once established, this production remains stable even after stress removal, i.e., when flies return to a normal temperature of 25 °C. Small RNA sequencing analyses were unable to identify other genomic loci used for de novo piRNA synthesis as if all loci competent to become piRNA cluster were already activated. Analyses performed at 29 °C to identify molecular factors required for the activation of de novo piRNA production suggest that there is an increase of transcription of the *BX2* cluster flanking region along with the transcription of a homologous euchromatic sequence. When *BX2* is maternally inherited, piRNA production is maintained, but when *BX2* is paternally inherited, piRNA production is abolished. This result suggests that when the stress is removed, piRNA can be self-maintained over generations by the paramutation process, as long as the inheritance is maternal [26] (Table 1).

In *Drosophila*, environmental stresses can generate piRNA emergence, and this new piRNA production is maintained across generations, allowing stable repression of homologous sequences. How can environmental stress impact piRNA production? It remains unclear whether piRNA cluster chromatin containing H3K9me3 marks, piRISC complex formation and/or stability, or piRNA pathway genes are involved in the stress response, individually or simultaneously. To date, it is quite difficult to estimate the global effect of piRNA deregulation over many generations, in particular, if it might result in the alteration of inherited gene expression patterns. Future investigations will help our understanding of environmentally-induced piRNA transgenerational inheritance and allow the estimation of potential consequences.

## 4. Speculative Insights on Transgenerational piRNA Inheritance Consequences

### 4.1. Implications in Evolution

According to Lamarck’s theory, the environment and the experiences of the organism mediate changes that could be inherited in subsequent generations. These changes are then subject to natural selection and adaptation [144,145]. In the case of transgenerational inheritance, the environment can impact the epigenome by causing epimutations, through DNA methylation, histone modifications, and non-coding RNA misregulation. These epimutations lead to phenotypic variations and can give the organism a selective advantage. In *C. elegans*, it has been shown that stress conditions (starvation, supplemented diet, salt, or arsenic) enhance longevity and stress resistance in subsequent generations [146,147,148]. If they are stable enough to be maintained over time, epigenetic modifications and their gradual accumulation in response to various stresses, may have an important role in speciation [149]. For Brevik and colleagues, transgenerational epigenetic inheritance may also explain the rapid emergence of insect population resistance against pesticides [150]. Many studies have shown that pesticides, notably vinclozolin or DDT, induce transgenerational changes in DNA methylation, histone modification, and/or non-coding RNA deregulation in rodent models (Table 1).

Among the epigenetic mechanisms driving adaptation and evolution, piRNAs are playing a fundamental role in TE regulation across generations. For instance, during the 20th century, the *D. melanogaster* genome was invaded by the *P* element after a horizontal transfer [151]. It appears that in the absence of *P* element regulation, progeny display genetic defects, leading to gonad atrophy at high temperature, whereas no anomaly occurs when *P* element regulation is established [152,153]. It was later shown that this specific control of gonadal *P* element occurs through piRNAs [101]. Interestingly, the *Drosophila simulans* genome has been recently invaded by *P* elements [154], and the invasion dynamics are widely influenced by temperature. The transposition rate at elevated temperature is higher compared to the rate at low temperature [155]. At elevated temperature (29 °C), after 20 generations, the *P* element copy number in the genome reaches a plateau. These invasions are correlated with piRNA production against *P* element sequences, strongly suggesting that the regulation is established in response to invasion.

Analyses of *P* element distribution in the *D. melanogaster* genome showed that subtelomeric regions of the *X* chromosome are hotspots for *P* element insertions [156]. Those subtelomeric regions happened to be one of the active *Drosophila* piRNA clusters [74]. Once *P* element or *P* derived transgenes insert into subtelomeres, new piRNAs targeting those sequences will be synthesized, leading to repression of euchromatic copies [71,101,114,116,157]. Particular sequences in subtelomeric piRNA clusters, called *TAS-L like* (*TLL*), found in the *X* chromosome of *D. melanogaster,* are also present in autosomic subtelomeric piRNA clusters of other members of the *melanogaster* subgroup, including *D. simulans*. The finding that some *P* element insertions were found very close to *TLL* in *D. simulans* suggests that *P* element regulation could be conserved between *D. melanogaster* and *D. simulans* [155,157]. As with all piRNA clusters, the subtelomeric regions are made up of repeated domains that were once activated for piRNA production. However, the type of event that converted these loci remains unclear. Recently, we have shown that high temperature can convert a locus composed of tandemly repeated sequences into a stable piRNA cluster, highlighting the sensitivity of such regions to environment stresses for piRNA production [26]. Once activated, de novo piRNA production can be transmitted through generations by maternal inheritance [71]. Because these loci are hotspots of insertion for TEs, specific piRNAs are synthesized that functionally repress homologous sequences, leading to maintenance of genome integrity over time. Therefore, piRNA clusters constitute an epigenetic memory of TE invasion and are essential to provide genome defense.

TEs contain many regulatory sequences, including insulators, RNA polymerase signals, splice sites, and/or environment response elements [158]. Furthermore, TEs can modulate the expression of neighboring genes [159]. In addition, numerous stresses, including radiation, pollutants, temperature, or viral infection, can induce TE activation [160,161]. Thus, similar to *P* element activity under elevated temperature conditions, the emergence of piRNA production can occur in response to stress-induced TE activation. We can expect that organisms with TE regulatory mechanisms are likely to be selected as compared to organisms without this defense system. Indeed, TE mobilization induced by environmental stress can affect genome organization by creating mutations, chromosomal breaks, or genomic rearrangements [162,163]. In the long-term, these modifications can be beneficial for organisms by the creation of new molecular functions, but until this occurs, TEs must be regulated in the short-term to restrict genomic damage. In a way, if TEs are defined as molecular actors of evolution, piRNAs could act as molecular regulators of evolution.

Finally, piRNAs and molecular factors of the piRNA pathway have been described to play a role in reproductive isolation by maintaining interspecies sterility [164,165]. In *D. melanogaster* and *D. simulans* hybrids, *D. simulans* Rhino protein co-localizes with *D. melanogaster* Deadlock, but they cannot interact, because of the rapid evolution of their chromo domains. As a consequence, TE mobilization leads to hybrid sterility due to the absence of a functional piRNA pathway [164]. Additionally, the *AT-chX* locus, located close to the pericentromeric region of the *X* chromosome, is one of the *D. melanogaster* piRNA clusters absent from other *Drosophila* species. This locus contains sequences related to the *vasa* gene, which is required for germ cell development. Due to imperfect complementarity, piRNAs derived from the *AT-chX* locus are not able to repress *vasa* expression in *D. melanogaster* germ cells. Surprisingly, the *AT-chX* locus shares a high level of complementarity with the *vasa* gene from closely related species to *D. melanogaster*, leading to a repression of *vasa* in interspecies hybrid testes [165]. These studies suggest that piRNAs could play an important role in speciation.

### 4.2. Potential Implications for Disease Development

Environmental stresses, such as pesticide and pollutant exposure, have been shown to drive epigenetic inheritance of obesity and many associated diseases, including diabetes, infertility, immune disorders, and polycystic ovarian syndrome in rat. These transgenerational effects are associated with the emergence of differential DNA methylation regions, which contain genes involved in several processes, such as signaling, metabolism, transcription, development, and transport [23,24,25,32,35,36,38,166,167,168].

#### 4.2.1. piRNA Pathway and Disease

To date, there is no evidence for piRNA-mediated transgenerational inheritance of disease risks. However, paramutation and small RNA inheritance could represent a part of ‘missing heritability’ in some rare diseases that genetic mutations cannot explain (discussed in [169,170]). Deregulation of piRNAs and PIWI proteins has been found in some diseases, including many types of cancer. Hence, piRNAs are considered as cancer biomarkers along with several circulating RNAs [171]. Transcriptome analyses highlight the deregulation of piRNAs in several malignant tumors [172,173,174,175]. For instance, piR-Hep1 is upregulated in a hepatocellular carcinoma cell line [173]. piR-Hep1 knockdown reduces cell motility and invasiveness, whereas piR-Hep1 overexpression strongly increases cell migration. Finally, the high expression of human PIWI proteins (PiwiL) has also been associated with various cancers. Interestingly, the expression level of PiwiL2 is correlated with tumors aggressiveness. Moreover, PiwiL2 knockdown decreases cellular migration, suggesting a role in invasion [176,177].

It also appears that PIWI proteins are involved in different cellular processes, such as stem-cell maintenance or cell differentiation, in the *Drosophila* germ line [178] and tumor cell proliferation through induction of *c-Myc* expression in human cells [179]. As a consequence, piRNA and PIWI protein deregulation could sustainably affect these essential processes and induce developmental defects. It is possible that piRNA deregulation could partly explain cases of family susceptibility to cancer. Indeed, if the piRNA pathway is impaired, TE regulation is affected, and genome integrity is compromised.

#### 4.2.2. TEs and Disease

In different models (*Drosophila*, mouse, zebrafish, and human cultured cells), *p53* mutation leads to TE derepression associated with a piRNA biogenesis defect, at least in *Drosophila* [180]. Accordingly, *p53*-derived cancers have been considered as ‘transposopathies’, diseases induced by TE mobilization resulting in genome instability [181]. However, even though TE insertions have been detected in several cases of cancers, it is still unclear whether they are the direct cause of cancer development or a consequence of another cellular defect causing cancer. For instance, in colon cancer, retrotransposon insertion into the *APC* tumor suppressor gene leads to its inactivation; in cancer cell lines, retrotransposon insertions are linked to long chimeric transcript emergence and thus, drive the expression of several genes [182,183]. Transcriptome-wide analyses of RNA-seq data for several cancer types and tumors samples identified TE-oncogene chimera transcripts, a phenomenon called a ‘TE onco-exaptation event’. Several TE onco-exaptation events lead to oncogene activation or overexpression. For instance, *AluJb*, which drives *LIN28B* expression, has been implicated in lung cancer. DNA methylation of the *AluJb* sequence leads to a decrease in *LIN28B* expression [184].

Other diseases are linked to TE activity, including hemophilia [185,186], psychiatric disorders [187], and Alzheimer’s disease [188]. In the particular case of Alzheimer’s disease, piRNA molecules seem to be more abundant in the brain and can be considered as a molecular marker of this disease [189]. Indeed, transcriptome analyses of samples isolated from human brain show that some piRNAs are upregulated in Alzheimer’s disease [190].

Altogether, control of TE expression, especially by piRNAs, might play a crucial role in genome stability. If impaired by stress, such as mutation or environmentally-induced piRNA biogenesis misregulation, aberrant gene expression can occur inducing cancer development or other diseases related to TEs in subsequent generations, leading to some of the unexplained family susceptibility.

## 5. Conclusions

There is increasing evidence of environmentally-induced epigenetic inheritance, and today we have a better understanding of their associated mechanisms. However, there are still many points in our understanding of the interaction between the environment and the epigenome that are lacking, in particular in metazoans. Three major mechanisms are usually reported as mediators of epigenetic inheritance, namely DNA methylation, histone modification, and non-coding RNAs.

One stress may cause multiple epigenetic changes. Among these changes, some of them are probably independent, whereas other modifications work together. Nature is a combination of numerous stressors including temperature, pressure variations, pollution, starvation, drought as well as competition for food, social stresses, and so on. It is unlikely that one stress can act only on one biological process, but even if this is the case, we can expect that the addition of all these stresses will impact several important pathways.

A balance between transgenerational epigenetic inheritance and epigenetic reprogramming occurs at each generation, and these processes are tightly regulated during development. Several factors are capable of tipping this epigenetic balance toward transgenerational inheritance, notably after stress exposure. piRNA molecules could be one of these factors. Molecular mechanisms of transgenerational piRNA inheritance have been partially studied, and some hypotheses to explain their maintenance have been proposed, which include other epigenetic mechanisms, such as chromatin modifications and several other potential factors discussed above. For the moment, piRNA function in environmentally-induced epigenetic inheritance has been little explored. TE activity is found in some diseases, and many environmental stresses are known to generate TE mobilization events. In this context, piRNA misregulation may have an important impact on genome integrity and structure. Changes in piRNA expression could also modify the regulation of several genes and influence the initiation and development of some diseases. Future work will surely improve our understanding of the piRNA transgenerational effect in response to environmental injury.

## Figures and Tables

**Table 1:** A list of environmentally-induced transgenerational epigenetic inheritance.

Stress	Model/tested tissues	Epigenetic modifications	Reference
**Pesticides and pollutants**			
**Vinclozolin**	Rat/Sperm	DNA methylation	[28,29]
		miRNA, piRNA, tsRNA	[30]
		DNA methylation, histone retention, miRNA, piRNA, tsRNA, and lncRNA	[20]
	Rat/Prostate cells	DNA methylation, miRNA, piRNA, tsRNA, and lncRNA	[23]
	Mouse/Primordial Germ Cells	miRNA	[31]
	Rat/Ovaries	DNA methylation, piRNA	[25]
**Dichlorodiphenyltrichloroethane (DDT)**	Rat/Sperm, testis, ovaries, kidney, prostate, whole organism	DNA methylation	[32]
	Rat/Sperm	DNA methylation	[33]
		DNA methylation, histone retention, miRNA, piRNA, tsRNA, and lncRNA	[21]
	Rat/Ovaries	DNA methylation, piRNA	[25]
	Rat/ovaries, sperm, prostate, kidney, whole organisms	DNA methylation	[22]
**Pesticide mixture (Permethrin and DEET), plastic mixture (bisphenol A and phthalates), dioxin and jet fuel hydrocarbon**	Rat/Ovaries	DNA methylation	[34]
	Rat/Sperm, Ovaries	DNA methylation	[35]
**Plastic mixture (bisphenol A and phthalates)**	Rat/Sperm, testis, prostate, kidney, ovaries, whole organism	DNA methylation	[36]
**Glyphosate**	Rat/Sperm, testis, prostate, kidney, ovaries, whole organism	DNA methylation	[24]
**Chlordecone**	Mouse/Testis	H3K4me3 modification	[37]
**Hydrocarbon (jet fuel JP8)**	Rat/Sperm, ovaries, kidney, prostate, whole organism	DNA methylation	[38]
**Bisphenol A**	Nematode/Germinal cells	H3K27me3 and H3K9me3 modifications	[39]
**Heavy metals (Cu, Cd, Cr, and Hg)**	Rice/Leaf	DNA methylation	[40]
**Traumatic stresses**			
**Maternal separation**	Mouse/Sperm, brain	DNA methylation	[41]
**Fear conditioning**	Mouse/Sperm	DNA methylation	[42]
**Diet**			
**Low protein diet**	Rat/Liver	DNA methylation	[43]
**Starvation**	Nematode/Whole organism	siRNA	[44]
**Feeding with bacteria expressing ds-RNA (GFP)**	Nematode/Germinal Cells	siRNA	[45]
**Avoidance of pathogenic bacteria**	Nematode/Whole organism	piRNA	[46]
**Alcohol**	Rat/Sperm, POMC neurons	DNA methylation	[47]
**Nitrogen deficiency**	Rice/Leaf	DNA methylation	[48]
**Drought**	Rice/Seed	DNA methylation	[49]
**Osmotic and Thermic stresses**			
**NaCl**	Daphnia/Whole organism	DNA methylation	[50]
**NaCl, Heat shock**	*Drosophila*/Eyes	Heterochromatin disruption	[51]
**High temperature (25 °C)**	Nematode/Whole organism	H3K9me3 modification	[52]
	Nematode/Whole organism	H3K9me3 modification and siRNA	[53]
	Nematode/Oocytes	siRNA	[54]
**High temperature (29 °C)**	*Drosophila*/Ovaries	H3K9me3 modification and piRNA	[26]
